# Morphological Composition Influences Redundancy, Complementarity and Ecological Relevance of Habitat Complexity Metrics in Simulated Coral Communities

**DOI:** 10.1002/ece3.72077

**Published:** 2025-08-29

**Authors:** Daphne Oh, Anna K. Cresswell, Damian P. Thomson, Michael Renton

**Affiliations:** ^1^ School of Biological Sciences The University of Western Australia Crawley Western Australia Australia; ^2^ Environment, Commonwealth Scientific and Industrial Research Organisation (CSIRO) Crawley Western Australia Australia; ^3^ The UWA Ocean Institute The University of Western Australia Crawley Western Australia Australia; ^4^ The Australian Institute of Marine Science (AIMS) Crawley Western Australia Australia; ^5^ School of Agriculture and Environment The University of Western Australia Crawley Western Australia Australia

**Keywords:** coral reef, correlation, fish, rugosity, shelter, structural complexity

## Abstract

Earth's most complex and biodiverse ecosystems are characterised by high habitat complexity. On coral reefs, habitat complexity is influenced by the diverse morphology and composition of hard corals, shaping reef structure and shelter provision for many species. Various metrics are used to quantify reef complexity, yet, it remains unclear how well these metrics capture ecological functions such as shelter provision. We used a published dataset of 13 distinct coral community types generated using a 3D functional–structural model to investigate the redundancy, complementarity and ecological relevance of 11 habitat complexity metrics (seven ecologically meaningful shelter metrics and four more general structural metrics). We were especially interested in the extent to which structural metrics predicted shelter metrics, potentially reducing the need for more complicated and direct shelter measurements. We used Pearson's correlations to compare metrics in (i) one pooled analysis from all community types, and (ii) 13 individual analyses for each community type. In the pooled analysis, structural metrics were strongly correlated, while the shelter metrics formed two distinct groups—‘pelagic’ and ‘benthic.’ Within these groups, the metrics were highly correlated indicating redundancy, but between groups, they showed weak correlations indicating complementarity. For individual community types, the redundancy or complementarity of these metrics varied with coral morphology. Structural metrics were useful predictors of shelter metrics for certain community types; for example, surface rugosity was a strong predictor of shelter volume for tabular and digitate coral communities but not for other communities. Fractal dimension was highly complementary to other metrics, but further investigation is needed to identify its ecological relevance. We highlight that there is no universal metric, and it is important to consider a range of suitable habitat complexity metrics and morphological community composition for ecosystems with morphologically distinct biogenic habitat formers.

## Graphical abstract

Our study provides ecological insights on the most suitable metrics for habitat complexity, highlighting redundancies to streamline data collection. We demonstrate the influence of community composition on metric effectiveness, offering recommendations for measuring reef complexity and developing community‐specific functions to predict ecologically meaningful metrics from historical data for monitoring, management and restoration.

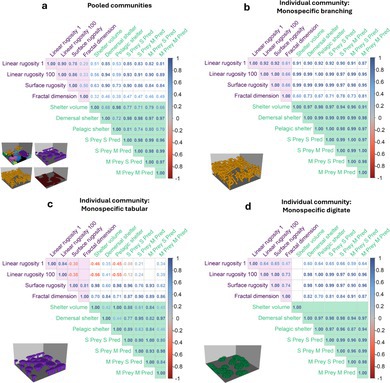

## 1 Introduction

The Earth's most biodiverse ecosystems, such as rainforests (Williams et al. 2002), kelp forests (Fulton et al. 2019) and coral reefs (Snelgrove et al. 2017), are all characterised by high habitat complexity. While the underlying geomorphology and topography contribute to this complexity, habitat structure is primarily shaped by the key habitat‐forming organisms such as trees (Romana et al. 2017), kelps (Teagle et al. 2017) and hard corals (Jones et al. 1994). By altering the overall physical structure, these organisms have the capacity to modify the biotic and abiotic components of their environments and are therefore termed ‘ecosystem engineers’ (Jones et al. 1994). These engineers create habitats across a wide range of spatial scales, influencing other species by modifying niche and resource availability (Hixon and Jones 2005; Luehrmann et al. 2020; O'Shea et al. 2013; Swisher et al. 1998) and shaping abiotic mechanisms such as wave attenuation and microclimate modification (Carlot et al. 2023; Milling et al. 2018; Zhang et al. 2022). These processes, in turn, dictate patterns of local species diversity, community structures and ecological functioning (Losapio et al. 2024; Sanders and Frago 2024; Schöb et al. 2012).

Scleractinian, or ‘hard’ corals, are the ecosystem engineers of tropical coral reefs. Their varied morphologies create habitat complexity that supports diverse assemblages of reef fish (Roberts and Ormond 1987; Sale 1977) and invertebrates (Idjadi and Edmunds 2006; Stella et al. 2010), while also contributing to coastal protection by buffering shorelines from wave energy (Sheppard et al. 2005). This complexity provides reef‐associated organisms with physical shelter from environmental stressors, competition and predation (Almany 2004; Beukers and Jones 1997; Kerry and Bellwood 2012), and is widely recognised as being tied to the many key ecosystem services that coral reefs provide (Bellwood et al. 2024).

Increasing disturbances have substantially degraded global coral reefs in recent decades (Bellwood et al. 2004; De'ath et al. 2012; Hughes et al. 2017), reducing their overall habitat complexity and likely their capacity to support diverse reef communities (Alvarez‐Filip et al. 2009; Emslie et al. 2014). Although the importance of habitat complexity on coral reefs is well established (Graham and Nash 2013; Roberts and Ormond 1987), we still have a limited understanding of how critical ecological processes, such as shelter from predation (Hixon and Beets 1993), are mediated by habitat complexity. This knowledge gap is partly due to challenges in comprehensively quantifying three‐dimensional reef complexity (Kovalenko et al. 2012) and the debates around which metric to use for this quantification. As coral reefs face ongoing declines, it is imperative to address these challenges to effectively evaluate changes in habitat complexity and the implications for reef functioning.

On coral reefs, habitat complexity has commonly been assessed using relatively simple metrics such as linear rugosity or percent coral cover (Alvarez‐Filip et al. 2011; Graham and Nash 2013). Linear rugosity is traditionally measured using the ‘chain‐and‐tape’ method (Luckhurst and Luckhurst 1978), where a chain is laid across the reef surface and the ratio of the contoured distance of the chain to a fixed linear distance is computed as an indicator of reef complexity (Bergman et al. 2000; Komyakova et al. 2013). Percent coral cover is typically estimated through visual assessments or measured using photo‐transects (Hill and Wilkinson 2004). The resulting percentage is then used as a proxy for habitat complexity where greater cover indicates greater complexity (Jones et al. 2004). Coral cover and linear rugosity are some of the most common indicators of reef health and condition due to the relative ease of data collection and analysis, and the global use over many decades. This is despite the recognition that these metrics are inadequate in effectively capturing processes crucial to reef functioning such as sub‐surface structural features like overhangs and crevices, which are likely important for providing shelter and influencing predator–prey interactions (Fakan et al. 2023; Helder et al. 2022).

In recent years, the use of structure‐from‐motion (SfM) photogrammetry for coral reef research has increased dramatically, producing scaled three‐dimensional (3D) reconstructions of reef surfaces (Burns et al. 2015). This has enabled the calculation of more detailed complexity metrics, such as ‘surface rugosity’ and ‘fractal dimension’ (Reichert et al. 2017; Torres‐Pulliza et al. 2020), with improved precision, accuracy and repeatability over the traditional chain‐and‐tape method and percent coral cover metrics (Barrera‐Falcon et al. 2021; Chen and Dai 2021; Lazarus and Belmaker 2021; Storlazzi et al. 2016). In addition, new metrics which estimate shelter provision, such as ‘shelter volume’ (Aston et al. 2022; Urbina‐Barreto et al. 2021), ‘viewshed’ (Aben et al. 2018; González‐Rivero et al. 2020; Oakley‐Cogan et al. 2020) and ‘size‐dependent shelter’ (Oh et al. 2025a), show considerable potential for describing species–habitat associations in reef fish (Agudo‐Adriani et al. 2016; Swanborn et al. 2022; Urbina‐Barreto et al. 2022). However, the unique contributions of different complexity and shelter metrics in capturing specific ecological functions remain unclear.

Empirical research on coral reefs presents significant challenges as hard corals, with their long lifespan and slow growth rates (Bythell et al. 2018), make tracking changes in coral community structure and their impacts on reef‐associated species over time difficult. Several studies have compared the efficiency and precision of conventional methods (e.g., chain‐and‐tape method; Luckhurst and Luckhurst 1978) to SfM photogrammetry (Curtis et al. 2023; Kornder et al. 2021; Monfort et al. 2021) since some are easier and more cost‐effective to measure than others. For example, while SfM photogrammetry can better capture 3D metrics like surface rugosity and shelter volume (Aston et al. 2022), this method is computationally challenging and can be costly (Bayley and Mogg 2020). In some cases, traditional manual methods (i.e., linear rugosity or visual assessments; Wilson et al. 2007) may be more feasible and affordable than photogrammetry as more complex and larger reef structures require longer processing time and more images (House et al. 2018; Wilson et al. 2007). Furthermore, long‐term monitoring data exist for the simpler metrics, and it is important to understand how these established methods compare to novel metrics. Efforts to convert easily obtained two‐dimensional (2D) metrics into ecologically meaningful attributes have shown promise at the scale of individual coral colonies (Aston et al. 2022; House et al. 2018; Urbina‐Barreto et al. 2021), but is more complicated at larger spatial scales across coral communities. Identifying redundancy and complementarity of complexity metrics, particularly for those that are practical and cost‐effective, is valuable for streamlining research and informing conservation efforts.

This study aimed to investigate the redundancy and complementarity of different habitat complexity metrics categorised into ‘structural’ metrics, which quantify general aspects of the physical structure of a coral community, and ‘shelter’ metrics, which more directly quantify the provision of shelter or refuge for reef species within a coral community. Here, ‘redundancy’ is defined as strong correlations between metrics, suggesting that the metrics are relatively interchangeable and do not provide different independent information about structural complexity and shelter. ‘Complementarity,’ in contrast, is defined by low or insignificant correlations, indicating that the metrics provide distinct information and thus can be used together to better capture different aspects of complexity and shelter. Specifically, we investigated:

1. Whether four structural metrics were redundant or offered independent and thus complementary information.
2. Whether seven different shelter metrics were redundant or complementary.
3. The extent to which structural metrics predicted shelter metrics, potentially reducing the need for more complicated direct shelter measurements.

## 2 Methods

For this study, we used a published dataset on three‐dimensional (3D) coral communities generated in a previous study (Oh et al. 2025a, 2025b). That study focused on coral cover and morphological diversity as predictors of habitat complexity, whereas here we reanalysed that data to address our novel aim of identifying the redundancy and/or complementarity among a range of habitat complexity metrics. The link to the dataset and R code used in the present study can be found in the data availability statement.

### 2.1 Model Description

The dataset was generated using *Coralcraft*, a model that simulates the dynamic 3D functional–structural growth and development of communities of various types of corals, accounting for competition for light and space in a 3D space (Cresswell et al. 2020; Oh et al. 2025a). The model represents a heterogeneous coral community within a 1 m^3^ virtual space with uniform physical and environmental conditions bounded by the seafloor at the bottom (*z* = 1) and the water surface at the top (*z* = 100) and a spatial resolution based on 1 cm^3^ ‘voxels.’. The horizontal edges of the simulation are ‘wrapped’ in a torus, eliminating edge effects and effectively simulating a larger homogenous area (as detailed in Cresswell et al. 2020). The model runs on a discrete weekly time step, corresponding to 52 time steps per year.

*Coralcraft* was especially suited to this study on the habitat complexity provided by live hard corals, as it provides a 3D representation of the structural development in coral communities over time. This allowed us to account for the structure and shelter contributed by individual coral colonies, as well as the spaces between corals, including overhangs and crevices that are difficult to capture empirically. The seafloor was assumed to be flat; thus, we were not attempting to account for any underlying reef geomorphology or other benthic components such as soft coral, macroalgae, or dead coral skeletons that may contribute to habitat complexity.

### 2.2 Coral Morphologies and Demographic Parameters

Ten distinct coral morphologies that broadly represent the range of common growth forms observed on coral reefs—encrusting, hemispherical, digitate, corymbose, tabular, mushroom, columnar, foliose, bushy and branching—were represented in *Coralcraft* (as described and illustrated in Oh et al. 2025a). Coral growth is restricted within the predefined morphologies, with colonies able to attain a maximum radius of 49 cm and height of 50 cm (see Cresswell et al. 2020). All morphologies have similar growth rates, except for the tabular morphologies, which grow at twice the rate to reflect their rapid growth (Pratchett et al. 2015; as detailed in Oh et al. 2025a). Coral morphological plasticity and species‐specific morphological variation in response to local environmental conditions were not considered (Chappell 1980; Paz‐García et al. 2015; Doszpot et al. 2019). However, spatial competition between colonies was still incorporated into the model, where adjacent corals can grow around one another depending on which colony occupies the space first.

### 2.3 Simulated Coral Communities

**FIGURE 1:**
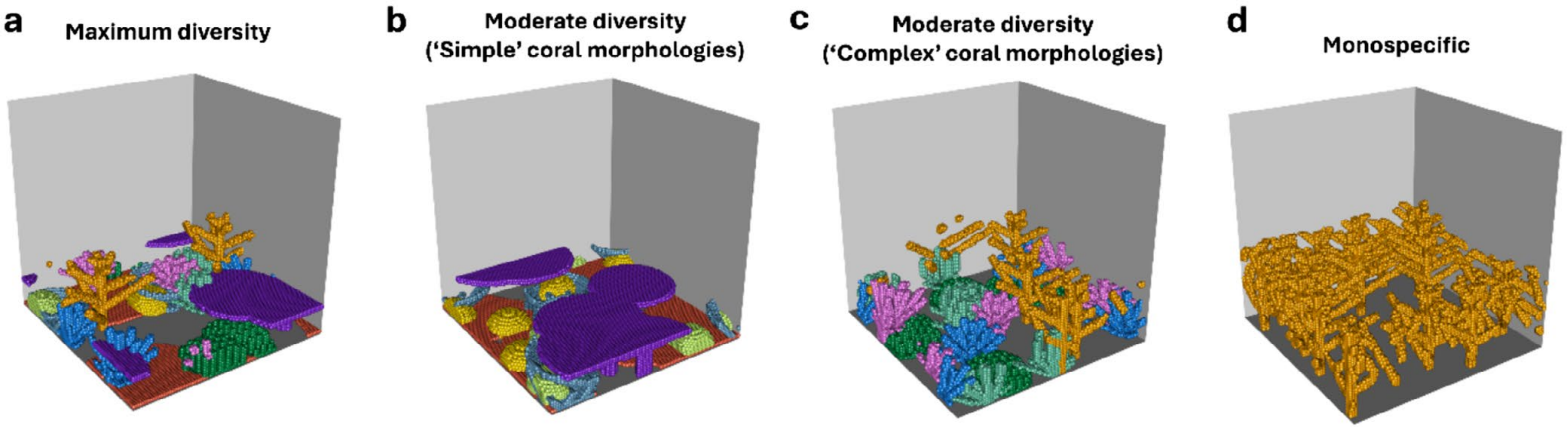
Examples of coral community types simulated in *Coralcraft*: (a) *Maximum diversity* consisted of all 10 coral morphologies, *Moderate diversity* consisted of (b) the five simplest morphologies (encrusting, hemispherical, tabular, mushroom and foliose) or (c) the five most complex morphologies (digitate, corymbose, bushy, corymbose and branching) and (d) 10 *monospecific* communities, each containing one morphology, here shown for a monospecific branching community.

We considered 13 distinct ‘coral community types’ with varying levels of morphological diversity and composition of 10 different coral morphologies (Figure 1; see figure 1 in Oh et al. 2025a for more detail). The growth of coral communities was simulated over 5 years from a post‐disturbance state with 20 individual coral colonies (at smallest possible size of 1 cm^3^) randomly placed on the bottom of the simulated area in the first time step. A total of 100 replicate simulations of each community type were conducted to capture stochasticity in the growth of communities dependent on the initial location of coral colonies.

### 2.4 Habitat Complexity Metrics

We examined 11 habitat complexity metrics (Table 1). Four were categorised as ‘structural’ metrics, describing the physical architecture of the coral community: (i) Linear rugosity sampled once across the centre of the coral community (hereinafter as ‘linear rugosity 1’), (ii) linear rugosity sampled 100 times across the community (hereinafter as ‘linear rugosity 100’), (iii) surface rugosity and (iv) fractal dimension. The next seven, we categorised as ‘shelter metrics,’ directly describing shelter for reef‐associated species: (v) shelter volume, shelter from (vi) demersal and (vii) pelagic predators and (viii–xi) four variations of predator and prey size‐dependent shelter (Oh et al. 2025a). Metrics were computed every 13 time steps for each community type for each of the 100 replicate simulations.

**TABLE 1:** Description and schematics of the 11 habitat complexity metrics and the method of calculation in *Coralcraft*. Structural metrics describe the physical architecture of a coral community, while shelter metrics describe the shelter provided to reef‐associated species within a coral community (adapted from Oh et al. 2025a).

Metric	Description	Calculation in *Coralcraft*
*Structural metrics*		
(i) Linear rugosity 1 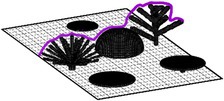 (ii) Linear rugosity 100 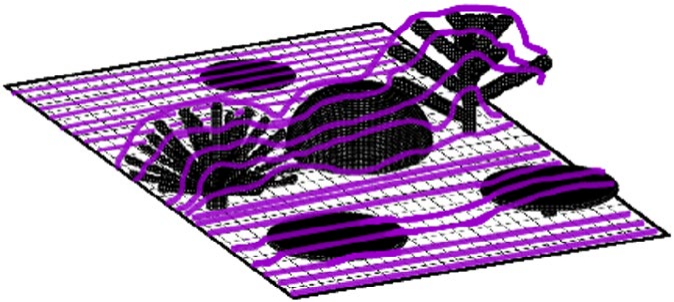	Most common structural complexity metric in coral reef research (Graham and Nash 2013) Analogous to the chain‐and‐tape method (Luckhurst and Luckhurst 1978) Value of 1 indicates a perfectly flat environment, whereas values higher than 1 indicate greater rugosity	Ratio calculated between the length of upward‐facing contours of the simulated coral community (as if laying a chain over the reef) and the horizontal linear distance across the environment (100 cm) Examined both at (i) a single *x* value, *x* = 50, and (ii) as mean of every *x*‐plane (i.e., the mean of 100 measures). (See Supporting Information for analysis of linear rugosity 10—mean of 10 measures)
(iii) Surface rugosity 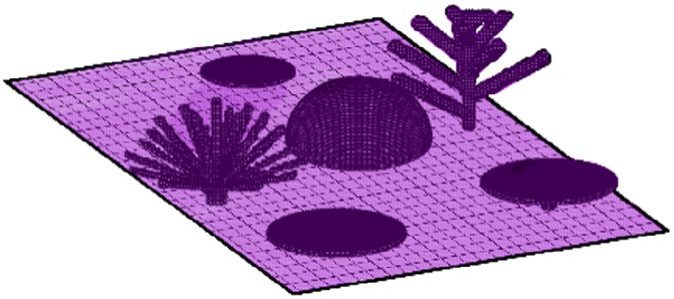	3D transposition of linear rugosity (Burns et al. 2015) Value of 1 indicates a perfectly flat environment whereas values higher than 1 indicate greater rugosity	Ratio calculated from the 3D surface area of all corals and any unoccupied seafloor versus the 2D planar area (100 × 100 cm)
(iv) Fractal dimension 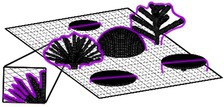	Captures how the fragmented spaces (i.e., microhabitats) created by corals fill the volume of a given space Values of FD are bounded between 2 (a completely flat plane) and 3 (a theoretical 2D surface completely filling a 3D space)	Calculated using the cube‐counting method outlined in (Zawada et al. 2019) The slope of log_10_(*N*) ~ log_10_(*C*), where *N* is the total number of voxels that contain any surface of coral voxels and *C* is the vector of standardised cube sizes (2, 4, 5, 10, 20 cm) used to calculate fractal dimension in the 3D array
*Shelter metrics*		
(v) Shelter volume 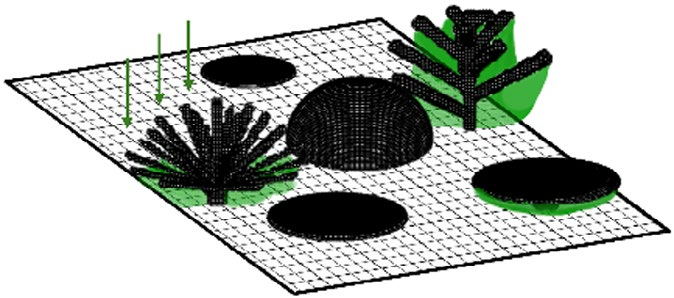	A volumetric measure of interstitial space within coral communities (Aston et al. 2022) Higher values indicate more shelter within the environment. Zero means no shelter	Total sum of 1 cm^3^ empty voxels directly covered by coral voxels above in the *z*‐axis
(vi) Demersal shelter 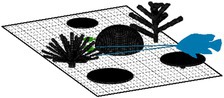	Estimates the visual exposure of a hypothetical prey from demersal predators hunting horizontally within coral communities (adapted from viewshed analysis; Aben et al. 2018) Higher mean percentage indicates greater degree of demersal shelter, representing greater shelter capacity within coral community	Mean percentage of 1 cm^3^ empty voxels covered by coral voxels when viewed from four vertical sides of the environment (north, south, east and west) This calculation assumes maximum vertical and horizontal predatory fields of view. Demersal shelter is determined solely by the complexity of coral structures and is not influenced by a combination of structural complexity and predator field of view
(vii) Pelagic shelter 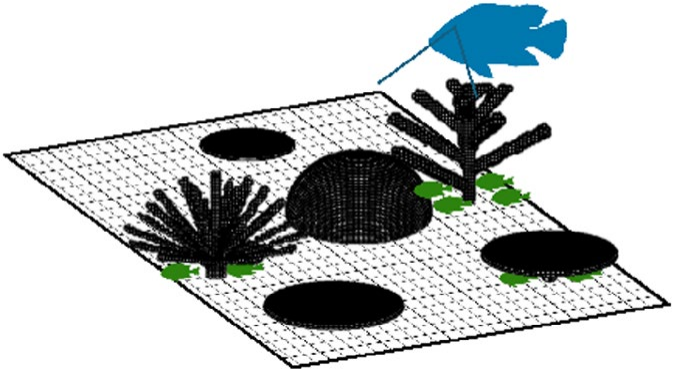	Estimates the visual exposure of a hypothetical prey to pelagic predators swimming above coral communities (adapted from viewshed analysis; Aben et al. 2018) Higher mean percentage indicates greater pelagic shelter	At a preset *z*‐value (height within the environment), the mean percentage of 1 cm^3^ empty voxels covered by coral voxels when viewed from five different viewpoints: directly above and at 45° angles to the north, south, east and west of the environment/seafloor is calculated Overall pelagic shelter is then measured as the mean percentage calculated from a range of *z*‐values (from *z* = 1 to *z* = 15)
(viii–xi) Size‐dependent shelter (4 variations, only two shown here) 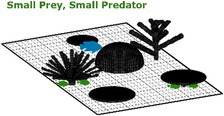 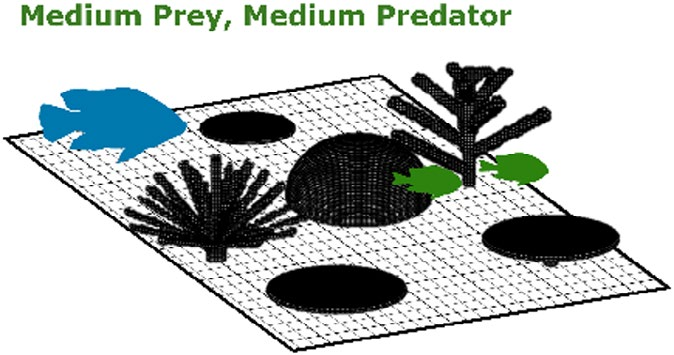	Estimates the space available for a small prey of a given body size seeking shelter from a larger predator	Measured as the mean percentage of empty voxels (i.e., interstitial spaces created by corals) where a prey of set size can occupy but a predator of set larger size cannot Examined for small and medium prey (set size of 1 and 3 cm) with small and medium predators (set size of 9 and 21 cm) with four varying combinations^a^

^a^ The size classes used in this study are representative of many coral‐associated fish species found in Indo‐Pacific coral reefs (Goatley et al. 2017; Brandl et al. 2018). Similar size classes are commonly used to examine predator–prey interactions (Holmes et al. 2012; Thillainath et al. 2016), with prey fish of those size classes relying on corals for shelter (Pratchett et al. 2008; Wilson et al. 2010). These studies highlight the relevance of these size classes in assessing fish populations and their interactions with coral habitats. However, this metric could also be calculated for any other size classes, depending on fish species of interest.

### 2.5 Statistical Analysis

We used Pearson's correlation tests to quantify the strength of correlation between the 11 habitat complexity metrics with two approaches to utilise the full dataset. First, we did a pooled analysis of all data across all community types (13 community types, 11 metrics measured every 13th time step, 100 simulations, *n* = 26,000 for each metric), to represent a wide variety of different types of coral communities at different stages of development (hereinafter as ‘pooled communities’). Second, we conducted 13 individual analyses for each community type (1 community type, 11 metrics measured every 13th time step, 100 simulations, *n* = 2000 for each metric), to represent specific types of coral communities that may be found on a reef at different stages of development (hereinafter as ‘individual communities’ or the specific community type, that is, ‘monospecific branching community’). Correlations are discussed as weak (|*r|* < 0.3), moderate (0.3 < *|r|* < 0.7), strong (0.7 < *|r|* < 0.9) or very strong (|*r|* > 0.9). To visualise and assess the relationships between different combinations of metrics, we fitted cubic models using the nlme package (Pinheiro et al. 1999) and applied principal component analysis (PCA) using the FactoMineR (Lê et al. 2008) and factoextra packages (Kassambara and Mundt 2016) in R (R Core Team 2024).

## 3 Results

### 3.1 Correlations Among Structural Metrics

**FIGURE 2:**
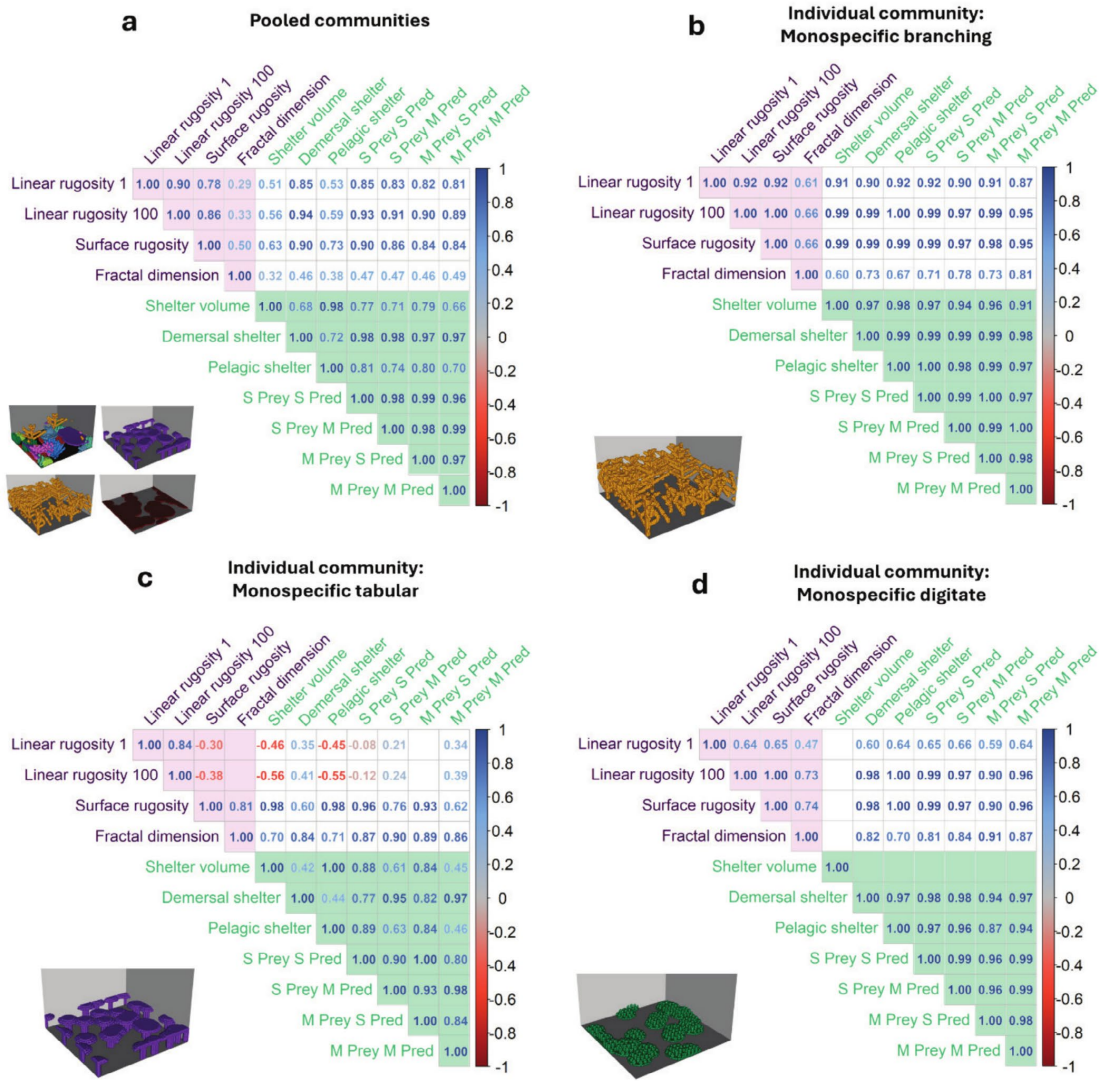
Correlation matrices among structural (purple text) and shelter (green text) metrics calculated every 13 time steps from 100 replicate simulations—(a) correlations for pooled communities, calculated across all 13 community types, and correlations for individual communities: Monospecific (b) branching, (c) tabular and (d) digitate community types, calculated separately. Numerical values represent the correlation value to two decimal places as calculated by Pearson's correlation test. The colour of the numbers indicate strength of correlation (red: Negative correlation, blue: Positive correlation, see colour gradient bar). Correlation value is only shown when significant (*p* < 0.05). Purple background shading indicates correlations among structural metrics, green background indicates correlations among shelter metrics and correlations between structural and shelter metrics have unshaded background. For similar results for other individual communities see Figure S1.

The structural metrics—the two measures of linear rugosity, surface rugosity and fractal dimension (purple shading in Figure 2)—showed varied strengths of correlations with each other when examined for pooled communities (Figure 2a) and individual communities (Figures 2b–d, S1).

Linear rugosity 1 and linear rugosity 100 were very strongly positively correlated in the pooled community analysis (*r* = 0.90; Figures 2a and S2), monospecific branching (*r* = 0.92; Figure 2b) and monospecific corymbose communities (*r =* 0.91; Figure S1f). Correlations were strong in other communities such as the monospecific tabular (*r* = 0.84; Figure 2c), the moderate‐diversity (complex) (*r* = 0.83; Figure S1c) and various monospecific communities (*r* > 0.8; Figure S1). This relationship was moderate in the monospecific digitate (*r* = 0.64; Figure 2d) and moderate‐diversity (simple) community (*r* = 0.69; Figure S1b).

Linear rugosity 1 and surface rugosity were strongly positively correlated in pooled communities (*r* = 0.78; Figures 2a and S2) and particularly so within the monospecific branching community (*r* = 0.92; Figure 2b). This relationship was more moderate in other individual communities, such as the monospecific digitate (*r* = 0.65; Figure 2d) and both moderate‐diversity communities (both *r* = 0.6; Figure S1), and was negative in the monospecific tabular community (*r* = −0.38; Figure 2c).

Linear rugosity 100 was also strongly positively correlated to surface rugosity in the pooled community analysis (*r* = 0.86; Figures 2a and S2), and very strongly positively correlated in each individual community (*r* > 0.9; Figures 2b,d and S1). Only the monospecific tabular community showed moderate and negative correlation between linear rugosity 100 and surface rugosity (*r* = −0.38; Figure 2c).

Correlations between fractal dimension and the other structural metrics were moderate across the pooled communities (0.29 ≤ *r* ≤ 0.50; Figures 2a and S2). Fractal dimension showed the most varied correlations with other metrics when considering individual communities (Figures 2b–d, S1). For example, in the monospecific foliose community, fractal dimension was strongly positively correlated with the other structural metrics (0.70 ≤ *r* ≤ 0.84; Figure S1), while in the monospecific branching (0.61 ≤ *r* ≤ 0.66; Figure 2b) and maximum diversity community (0.50 ≤ *r* ≤ 0.68; Figure S1) it was moderately correlated with the other structural metrics. The correlations between fractal dimension and the two linear rugosity metrics within the monospecific tabular community were insignificant (*p* > 0.05; Figure 2c).

### 3.2 Correlations Among Shelter Metrics

Two distinct groupings were observed among the shelter metrics (green shading in Figure 2) in the pooled community analysis (Figures 2a and S2): shelter volume was very strongly correlated with pelagic shelter (*r* = 0.98), and demersal shelter and the four size‐dependent shelter metrics were all very strongly correlated with each other (*r* ≥ 0.96). The correlations between metrics across these two groups were weaker (0.55 ≤ *r* ≤ 0.81; Figure 2a). This indicates a ‘pelagic’ grouping (pelagic shelter and shelter volume) and a ‘benthic’ grouping (demersal shelter and the four size‐dependent shelter metrics). Similar ‘pelagic’ and ‘benthic’ groupings were observed when considering the monospecific tabular community (Figure 2c). While shelter volume was even more strongly grouped with pelagic shelter (*r* = 1.00) for the tabular community, the ‘benthic’ grouping was less consistent, with the two small‐predator size‐dependent metrics more correlated with pelagic shelter than demersal shelter.

In some individual communities (i.e., monospecific branching, bushy, columnar, foliose, corymbose community and the maximum diversity community; Figures 2b and S1), all shelter metrics were very strongly correlated with one another (*r* > 0.90). This did not hold for other individual communities such as monospecific digitate, hemispherical and mushroom communities, where shelter volume showed insignificant or weak correlations with other shelter metrics (Figures 2d and S1).

### 3.3 Relationships Between Structural Metrics and Shelter Metrics

**FIGURE 3:**
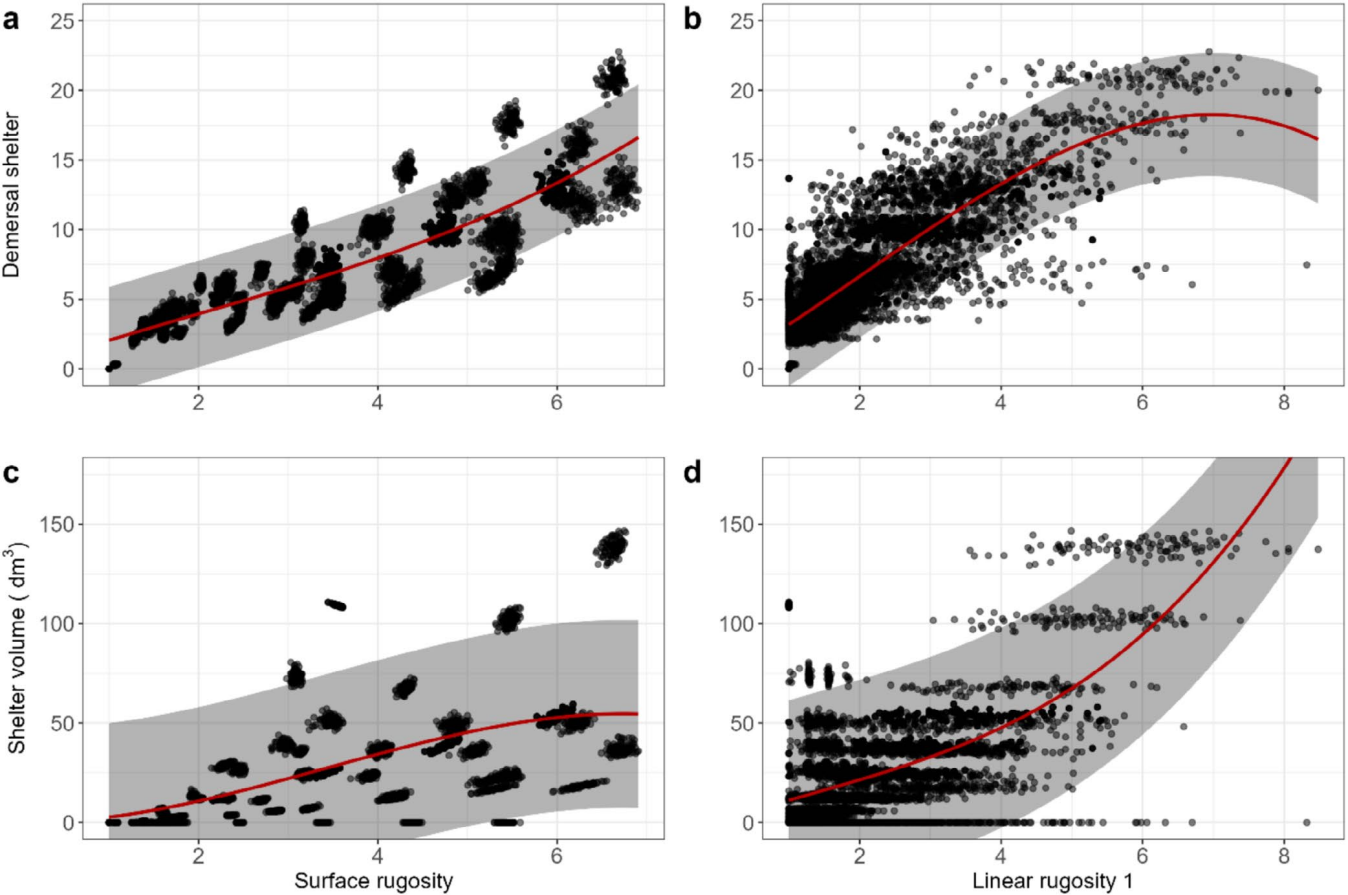
Relationships between two structural (surface and linear rugosity 1, *x*‐axis) and two shelter (shelter volume and demersal shelter, *y*‐axis) metrics calculated every 260 time steps for all 100 simulations of data pooled together across 13 community types. Red line and grey ribbon show the mean and 95% prediction interval from statistical cubic model added to aid pattern visualisation.

When considering pooled communities, all correlations between structural and shelter metrics were significant and at least moderately positive (*r* > 0.30; unshaded squares in Figure 2a). Fractal dimension was only moderately correlated with the shelter metrics (0.32 ≤ *r* ≤ 0.49; Figures 2a and S2). The other structural metrics were more correlated with the shelter metrics in the previously identified ‘benthic’ group (*r* > 0.80) than in the ‘pelagic’ group (0.51 ≤ *r* ≤ 0.73). Notably, surface rugosity was more closely grouped with the ‘benthic’ group than the two linear rugosity metrics (Figure S2). The shape of these relationships varied (Figures 3 and S3); in some cases, shelter metrics increased in a relatively linear manner with increasing structural complexity (Figure 3a), and in other cases more logarithmically (Figure 3b,c) or exponentially (Figure 3d).

When considering the relationships for each individual community, correlations between the structural and shelter metrics were mostly significant and at least moderately positive (unshaded in Figures 2b–d, S1). The monospecific tabular community was a clear exception (Figure 2c), with significant negative correlations between the two linear rugosity structural metrics and the two ‘pelagic’ shelter metrics (*r* < −0.40), and weaker (*r* < −0.20) or nonsignificant correlations with other shelter metrics. For certain individual communities such as monospecific tabular and digitate communities (Figure 2c,d), fractal dimension was more correlated with shelter metrics than the other structural metrics, which was contrary to pooled communities (Figure 2a). In other cases, such as the monospecific corymbose and columnar communities, fractal dimension was less correlated with shelter metrics compared with other structural metrics, similar to the pooled communities (Figure S1). Unlike in pooled communities, there was no clear pattern of structural metrics being more correlated with ‘benthic’ than ‘pelagic’ shelter metrics for individual communities (Figures 2b–d, S1). Additionally, structural metrics were less, or not at all, correlated with the shelter volume metric in individual communities such as the monospecific digitate (Figure 2d), hemispherical and mushroom (Figure S1), a similar pattern to that observed in pooled communities.

**FIGURE 4:**
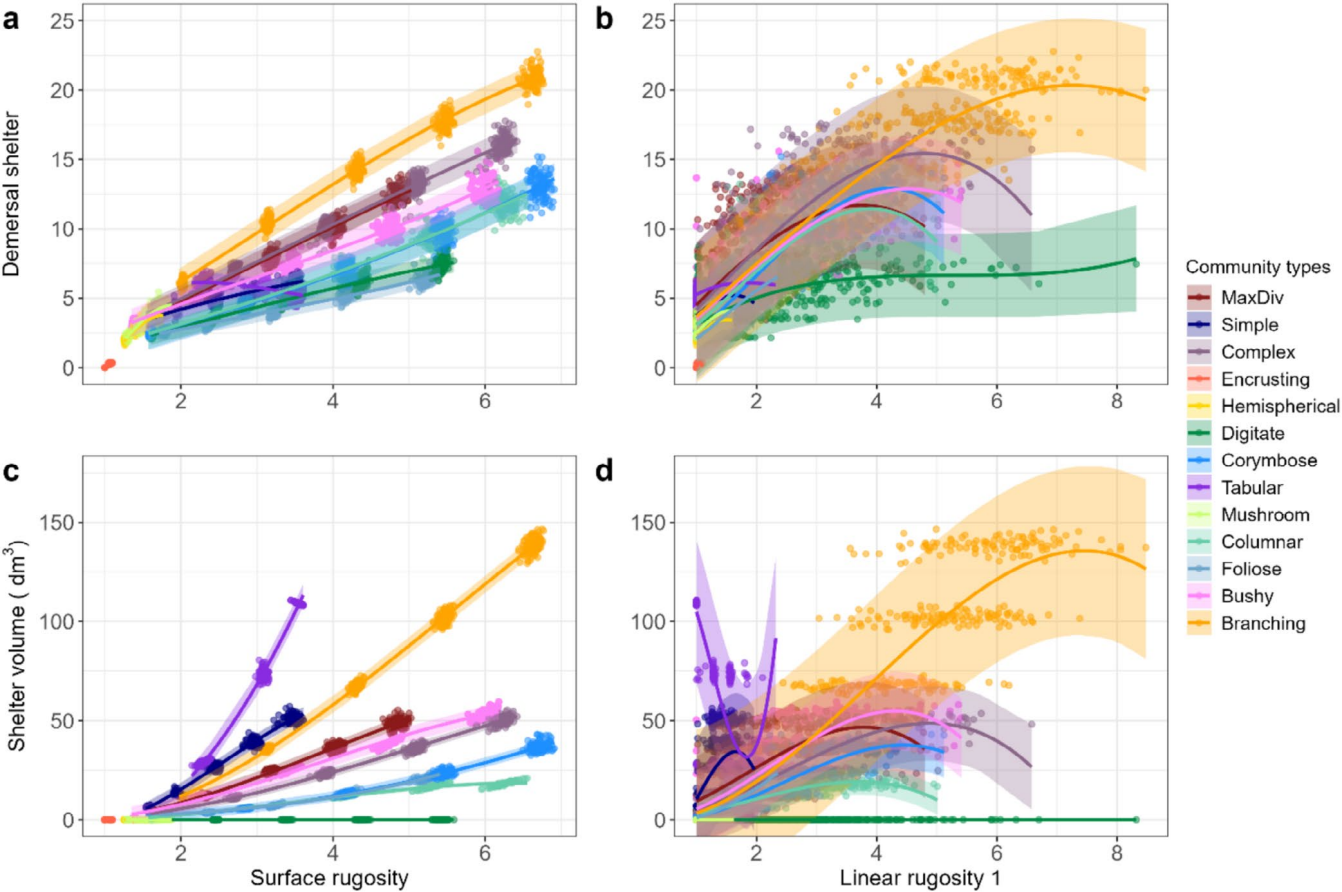
Relationship between two structural (surface and linear rugosity 1, *x*‐axis) and two shelter (shelter volume and demersal shelter, *y*‐axis) metrics calculated every 260 time steps across all 100 replicates of each of the 13 community types. Coloured lines and ribbons show the mean and prediction interval from the statistical cubic models of the respective community type to aid pattern visualisation.

There were varying degrees of nonlinearity in the curve predicting the mean value of shelter metrics from structural metrics, with linear, logarithmic and exponential shaped positive relationships observed similar to those in the pooled community analysis (Figures 4 and S4). There were also cases of U‐shaped relationships where a peak was followed by a decline, such as the relationship between linear rugosity 1 and demersal shelter for several community types (Figure 4b). Clear relationships between surface rugosity and the shelter metrics emerged, with little variation around the mean prediction and thus little overlap between individual communities (Figures 4a,c and S4). These relationships were particularly distinct for the two metrics in the 'pelagic' group (shelter volume and pelagic shelter, e.g., Figure 4c). The variation around the mean prediction was much smaller for relationships between surface rugosity and the shelter metrics (Figures 4a,c and S4), compared to those between linear rugosity 1 and shelter metrics (Figures 4b,d and S4). There was also much less variation around the mean prediction when considering individual communities than when considering pooled communities, when using surface rugosity as a predictor of both shelter metrics (Figure 4a,c; cf. Figure 4a,c).

## 4 Discussion

Developing predictive models that link structural complexity metrics to more directly ecologically meaningful metrics such as shelter provision across different coral community types is critical for enhancing the interpretation of historical data and improving understanding of habitat utilisation by reef‐associated species. Here, we identify redundant and complementary metrics to improve data collection efficiency by eliminating overlap and guiding the selection of cost‐effective structural metrics that predict key ecological attributes like refuge provision.

### 4.1 The Redundancy or Complementarity of Structural Metrics

The four structural metrics—particularly the rugosity metrics: linear rugosity 1, linear rugosity 100 and surface rugosity—were generally strongly correlated within and across community types, suggesting that these metrics are measuring structural complexity similarly to one another. However, we found inconsistency and high variability in the linear rugosity 1 metric, supporting concerns about the lack of reliable repeatability and precision in these linear rugosity 1 measurements (Leon et al. 2015). Our linear rugosity 1 metric closely reflects the ‘chain‐and‐tape’ method used in many coral reef studies (Castaño et al. 2021; Friedlander and Parrish 1998; Komyakova et al. 2013; Luckhurst and Luckhurst 1978; Risk 1972; Walker et al. 2009), where it has been used across scales from 2 m to tens of metres with no standardised distance of measurement. We considered linear rugosity in two ways: A single 1 m measurement across the middle (linear rugosity 1) and the mean of 100 parallel 1 m measurements across the entire simulated environment (linear rugosity 100). Given that these simulated rugosity measurements were taken within a spatially homogeneous area where the edges of the modelled environment are ‘wrapped’, our linear rugosity 100 metric is analogous to 100 more widely spaced replicate 1 m measurements or 10 replicate 10 m measurements or even one 100 m measurement. The fact that linear rugosity 100 was much less variable than linear rugosity 1 implies the benefit of measuring rugosity through replication or longer measurements to achieve more consistent estimates. In reality, however, measuring linear rugosity with a single chain over larger distances (such as 100 m) will likely introduce more variation due to changes in the underlying geomorphology, reef matrix and coral community types across this distance. Longer chains used in the chain‐and‐tape method are also logistically heavier and tangle easily. Therefore, replicate measures over shorter distances within smaller areas may be the most effective way to reliably measure the rugosity of a coral community. The particularly strong correlation between linear rugosity 100 and surface rugosity, a metric often obtained through photogrammetry (Ferrari, Bryson, et al. 2016; Magel et al. 2019; McCarthy et al. 2022), indicates that sufficiently reliable measures of linear rugosity can yield comparable precision and reliability to photogrammetrically derived structural metrics. While our results suggest 100 replicate 1 m measurements can ensure reliability, we also found that averaging just 10 linear rugosity 1 samples reduced variability to levels comparable to those obtained from the linear rugosity 100 metric (see Figure S5). This suggests that 10 replicate 1 m rugosity samples may be sufficient in practice, although this will likely depend on the area being sampled, the complexity of the coral community and underlying geomorphology. We can conclude that linear rugosity generally remains a useful index for structural complexity, particularly in morphologically diverse coral communities where photogrammetric techniques are challenging (e.g., available equipment/resources, water clarity) and the underlying reef structure difficult to photograph (Bayley and Mogg 2020). The metric can provide more robust estimates with repeated sampling to improve precision and to address issues of inconsistency (Storlazzi et al. 2016). However, the negative correlation between linear rugosity and surface rugosity observed for the monospecific tabular community reminds us that the relationship between linear and surface rugosity could be quite different in certain coral communities.

Fractal dimension is a mathematically well‐defined metric increasingly used in reef studies (Basillais 1997; Reichert et al. 2017; Torres‐Pulliza et al. 2020; Zhou and Lam 2005); however, the ecological mechanisms by which it may influence reef species across different spatial scales are uncertain. Fractal dimension emerged as a complementary metric to surface rugosity, showing consistently low correlations for most coral communities. This contrasts with previous studies that found strong correlations between fractal dimension and other 3D habitat descriptors (Fukunaga et al. 2019; Urbina‐Barreto et al. 2022). This discrepancy likely reflects differences in spatial scales, as our study focused on coral communities at centimetres to metres scales instead of larger reef areas of hundreds of metres (Burns et al. 2015; Casella et al. 2017). Structural complexity metrics vary in their ability to characterise reef structures across scales (Ferrari, McKinnon, et al. 2016; Fukunaga et al. 2020; Richardson et al. 2017), with previous research finding fractal dimension and surface rugosity useful for large reef areas (Torres‐Pulliza et al. 2020) while metrics like vector dispersion were more suitable for capturing fine‐scale variations in individual coral morphology (Fukunaga and Burns 2020) and overhanging surfaces in smaller reef areas (4 m^2^) (Young et al. 2017). Although fractal dimension had previously been suggested as a reliable measure of habitat complexity for larger areas of coral reef habitats (Fukunaga et al. 2019, 2020) and as an efficient method in quantifying the shape of individual coral colonies (Reichert et al. 2017), its ecological relevance at a local coral community scale remains unclear due to weak correlations with species richness (Beck 1998; Torres‐Pulliza et al. 2020). The poor correlations with other metrics investigated here suggest that fractal dimension may be capturing distinct aspects of complexity previously unmeasured at local scales, such as the diversity of shelter sizes and shapes (Zawada et al. 2019), which could represent ecological mechanisms not described by the seven shelter metrics.

### 4.2 The Redundancy or Complementarity of Shelter Metrics

We identified two distinct groups of shelter metrics, where metrics within a group were highly correlated but metrics in different groups were not. The strong correlations within the ‘pelagic’ group (shelter volume and pelagic shelter), as well as within the ‘benthic’ group (demersal shelter and size‐dependent shelter), suggest redundancy and that little additional information would be obtained in measuring both metrics for most coral communities. Conversely, the low correlations between the ‘pelagic’ and ‘benthic’ groups indicate they capture complementary information, offering potentially meaningful ecological insights into fish–habitat associations. For example, more structurally complex reefs create more enclosed spaces, reducing visual exposure to predators (quantified with demersal and pelagic shelter metrics) and providing more physical refuge from attacks (measured with shelter volume and size‐dependent shelter metrics), which have been shown to influence the abundances of site‐attached fish species (González‐Rivero et al. 2017; Harborne et al. 2012; Rilov et al. 2007). Different fish groups have displayed variable responses to differences in the habitat complexity of reefs attributed to the composition of coral morphologies (Darling et al. 2017; Nanami et al. 2005), where the shelter volume provided by each coral morphology influenced the fish composition (Urbina‐Barreto et al. 2022). Our findings collectively support those of Helder et al. (2022) and Lazarus and Belmaker (2021), who showed that no single habitat metric was uniformly important for predicting fish community compositions and abundance at local reef scales (25 m^2^), and instead different fish functional groups respond differentially to both large and small‐scale reef complexity indicators (Harborne et al. 2012; Helder et al. 2022; Urbina‐Barreto et al. 2022).

We found that coral community type influenced the degree of correlation between shelter metrics. In monospecific tabular coral communities, the ‘benthic’ and ‘pelagic’ metric groups were weakly correlated, suggesting they might provide complementary insights into predator–prey interactions. In contrast, these metric groups were highly correlated in monospecific branching and digitate coral communities, indicating redundancy. Predator–prey dynamics are shaped by coral morphology and substrate complexity, as larger predatory reef fish (e.g., *Lutjanidae*, *Haemulidae*, *Serranidae*) are often 'heavily reliant' on tabular corals for concealment and ambush (Kerry and Bellwood 2012, 2015), while smaller site‐attached prey fish (e.g., *Pomacentridae*) seek refuge in the dense branches of structurally complex corals (Nemeth 1998; Wilson et al. 2008). These findings highlight the importance of selecting complementary shelter metrics that align with coral community types, to best capture the ecological functions of habitat complexity for different fish groups.

The four size‐dependent shelter metrics were generally redundant, as illustrated by their strong correlations, suggesting that coral communities that provide shelter for larger prey against a given predator size are also likely to provide suitable shelter for smaller prey. Similarly, if a coral community cannot provide shelter for smaller prey, it may also be inadequate for larger prey. However, since these metrics have only been tested in simulation modelling (Oh et al. 2025a), empirical validation is essential to determine their reliability and ecological significance in real‐world coral reef environments. The correlation among these size‐dependent metrics calls for the development of a single metric capturing the abundance and size diversity of shelter spaces; exploring the relationship of such a metric with size‐specific fish abundance and diversity could provide valuable future insights.

### 4.3 Can Structural Metrics Be Useful Predictors of Shelter?

While strong positive correlations between the structural complexity and shelter provision have often been assumed (Bell and Galzin 1984; Sano et al. 1987), our results show that these relationships are not always linear, with both the strength and nature of the relationship varying significantly across different coral community types. We found that structural metrics can reliably predict shelter metrics when coral community type is known. For example, while surface rugosity was generally better than linear rugosity at predicting shelter (Figure 3), its accuracy improved when the coral community type was considered (Figure 4). More specifically, without knowing community composition, a surface rugosity of 4 would predict an estimate of shelter volume ~35 dm^3^. However, if community type was known, the estimate would be ~125 dm^3^ for tabular‐dominated communities and ~15 dm^3^ for corymbose or columnar‐dominated communities (Figure 4c). This is consistent with previous studies where incorporating coral colony morphology improved 3D complexity predictions (House et al. 2018), and accounting for coral community composition improved the hindcasting and predicting of the habitat complexity of coral reefs (Carlot et al. 2023).

Shelter provision may be predicted using functions derived from structural metrics, with the accuracy improved and variability reduced if the composition of the coral community is included in the function. While we demonstrate this for simulated coral communities, refining these relationships using empirical data is recommended. This would be beneficial in coral reef research for two reasons: structural metrics are currently easier to measure than shelter metrics, and historical data using metrics such as linear rugosity could be interpreted in terms of shelter metrics. However, it is important to note that singular measurements of the linear rugosity metric were highly variable, particularly when the community type was not considered, which might lead to inaccurate estimates of shelter provision. We found that in monospecific tabular and digitate coral communities, linear rugosity gave a poor representation of shelter while other 3D structural metrics such as surface rugosity and fractal dimension were better predictors. Notably, tabular‐dominated communities often have lower measurable structural complexity when assessed by linear rugosity, even when coral cover is high (Oh et al. 2025a), yet, these communities provide high levels of shelter and thus are of high ecological importance for reef species (Pascoe et al. 2021; Urbina‐Barreto et al. 2022). This highlights the importance of considering the high variability of the linear rugosity metric, and as such, depending on the coral community type, employing the use of a range of suitable complexity metrics to predict shelter metrics within coral communities. It is likely that previous applications of linear rugosity have not captured the true magnitude of habitat complexity on coral reefs, particularly when comparing varying states of reef degradation across different coral communities.

## 5 Conclusion

We conclude that there is no universal metric that captures all important aspects of habitat complexity for all coral communities. Therefore, it is important that future assessments of coral communities consider multiple suitable habitat complexity metrics and account for the morphological composition of coral communities to better capture structural complexity and shelter. This study is an important step in identifying the redundancies and complementarities of habitat complexity metrics, which can help streamline and improve efficiency in data collection and analysis while optimising resource allocation to assess both structural complexity and shelter provision of a coral community. Accounting for the type of coral community, specifically its morphological composition, will further inform the appropriate metrics to utilise, and can also aid in predicting meaningful ecological information such as refuge provision from easy‐to‐measure, cost‐effective structural metrics. Over time, we expect this to improve our understanding of reef fish–habitat relationships through more accurate and efficient quantification of habitat complexity and how that contributes to the heterogeneity in species distribution within coral reefs. Future research should prioritise replicating the present analysis on empirical observations of existing and newer habitat complexity metrics, focusing on the development of functions predicting shelter from structural complexity metrics for different coral community types using empirical data. These functions could then be applied to further interpret historical time series of ‘traditional’ structural complexity metrics, such as linear rugosity, further adding significant ecological value to historical data. We expect our findings here to be relevant in other ecosystems where the morphological community composition of biogenic habitat formers may affect the ecological relevance of habitat complexity metrics.

## Author Contributions

**Daphne Oh:** conceptualization (equal), data curation (lead), formal analysis (lead), methodology (equal), visualization (lead), writing – original draft (lead). **Anna K. Cresswell:** conceptualization (equal), formal analysis (equal), methodology (equal), supervision (equal), writing – review and editing (equal). **Damian P. Thomson:** conceptualization (equal), formal analysis (supporting), supervision (equal), writing – review and editing (equal). **Michael Renton:** conceptualization (equal), formal analysis (supporting), methodology (equal), supervision (equal), writing – review and editing (equal).

## Conflicts of Interest

The authors declare no conflicts of interest.

## Supporting information


**Figure S1:** Correlation matrices among structural (purple text) and shelter (green text) metrics calculated every 13 timesteps from 100 replicate simulations—correlations calculated for individual community types of (a) maximum diversity, moderate diversity with (b) simple and (c) complex coral morphologies, monospecific (d) encrusting, (e) hemispherical, (f) corymbose, (g) mushroom, (h) columnar, (i) foliose and (j) bushy community types. Numerical values represent the correlation value to two decimal places as calculated by Pearson's correlation test. The colour of the numbers indicates the strength of correlation (red: negative correlation, blue: positive correlation, see colour gradient bar). Correlation value is only shown when significant (*p* < 0.05). Purple background shading indicates correlations among structural metrics, green background indicates correlations among shelter metrics, and correlations between structural and shelter metrics have unshaded background.
**Figure S2:** Principal component analysis (PCA) ordination of (a) 11 habitat complexity metrics and (b) individual observations calculated every 260 time steps for all 100 simulations of data pooled together across 13 community types. Purple points and text indicate the four structural metrics, green points and text indicate the seven shelter metrics. See Oh et al. 2025a for detailed description of different coral community types.
**Figure S3:** Relationship between four structural (linear rugosity 100, fractal dimension, surface and linear rugosity 1, *x*‐axis) and four shelter (shelter volume, demersal shelter, pelagic shelter and 1 combination of size‐dependent shelter, *y*‐axis) metrics calculated every 260 time steps for all 100 simulations of data pooled together across 13 community types. Red line and grey ribbon show the mean and prediction interval from statistical cubic model added to aid pattern visualisation.
**Figure S4:** Relationship between four structural (linear rugosity 100, fractal dimension, surface and linear rugosity 1, x‐axis) and four shelter (shelter volume, demersal shelter, pelagic shelter and 1 combination of size‐dependent shelter, y‐axis) metrics calculated every 260 time steps across all 100 replicates of each of the 13 community types. Coloured lines and ribbons show the mean and prediction interval from the statistical cubic models of the respective community type to aid pattern visualisation.
**Figure S5:** Relationships between three linear rugosity metrics: (a) Linear rugosity 1 and linear rugosity 10 (mean of ten linear rugosity 1), (b) linear rugosity 1 and linear rugosity 100 and (c) linear rugosity 10 and linear rugosity 100 for all 100 simulations of data pooled together across 13 community types. Red line and grey ribbon show the mean and 95% prediction interval from statistical cubic model added to aid pattern visualisation.

## Data Availability

This study uses a published dataset on 3D coral communities generated in a previous study (Oh et al. 2025a), which can be found in the Zenodo Digital Repository https://doi.org/10.5281/zenodo.15074032. Coralcraft simulation code used in this study was adapted from the code in https://github.com/A‐K‐Cresswell/Coralcraft. The data and simulation code used in the present study can be found here: https://doi.org/10.5281/zenodo.16395986.
