# Supplementary material for: Morphological Composition Influences Redundancy, Complementarity and Ecological Relevance of Habitat Complexity Metrics in Simulated Coral Communities
Source: Ecol Evol. 2025 Aug 29;15(9):e72077. doi: 10.1002/ece3.72077 (PMC12395184; doi:10.1002/ece3.72077)
Supplement: Supplementary file 1 — Figure S1: Correlation matrices among structural (purple text) and shelter (green text) metrics calculated every 13 timesteps from 100 replicate simulations—correlations calculated for individual community types of (a) maximum diversity, moderate diversity with (b) simple and (c) complex coral morphologies, monospecific (d) encrusting, (e) hemispherical, (f) corymbose, (g) mushroom, (h) columnar, (i) foliose and (j) bushy community types. Numerical values represent the correlation value to two decimal places as calculated by Pearson's correlation test. The colour of the numbers indicates the strength of correlation (red: negative correlation, blue: positive correlation, see colour gradient bar). Correlation value is only shown when significant (p < 0.05). Purple background shading indicates correlations among structural metrics, green background indicates correlations among shelter metrics, and correlations between structural and shelter metrics have unshaded background. Figure S2: Principal component analysis (PCA) ordination of (a) 11 habitat complexity metrics and (b) individual observations calculated every 260 time steps for all 100 simulations of data pooled together across 13 community types. Purple points and text indicate the four structural metrics, green points and text indicate the seven shelter metrics. See Oh et al. 2025a for detailed description of different coral community types. Figure S3: Relationship between four structural (linear rugosity 100, fractal dimension, surface and linear rugosity 1, x‐axis) and four shelter (shelter volume, demersal shelter, pelagic shelter and 1 combination of size‐dependent shelter, y‐axis) metrics calculated every 260 time steps for all 100 simulations of data pooled together across 13 community types. Red line and grey ribbon show the mean and prediction interval from statistical cubic model added to aid pattern visualisation. Figure S4: Relationship between four structural (linear rugosity 100, fractal dimension, [file ECE3-15-e72077-s001.docx]

# Supplementary materials for

**Morphological composition influences redundancy, complementarity, and ecological relevance of habitat complexity metrics in simulated coral communities**

*Ecology and Evolution*

Daphne Oh^1,2,3^, Anna K. Cresswell^3,4^, Damian P. Thomson^2^, Michael Renton^1,5^

^1^ School of Biological Sciences, The University of Western Australia, Crawley, WA, Australia

^2^ Environment, Commonwealth Scientific and Industrial Research Organisation (CSIRO), Crawley, WA, Australia

^3^ The UWA Ocean Institute, The University of Western Australia, Crawley, WA, Australia

^4^ The Australian Institute of Marine Science (AIMS), Crawley, WA, Australia

^5^ School of Agriculture and Environment, The University of Western Australia, Crawley, WA, Australia

**Corresponding author**: Daphne Oh, [daphne.xy.oh@outlook.com](mailto:daphne.oh@research.uwa.edu.au)


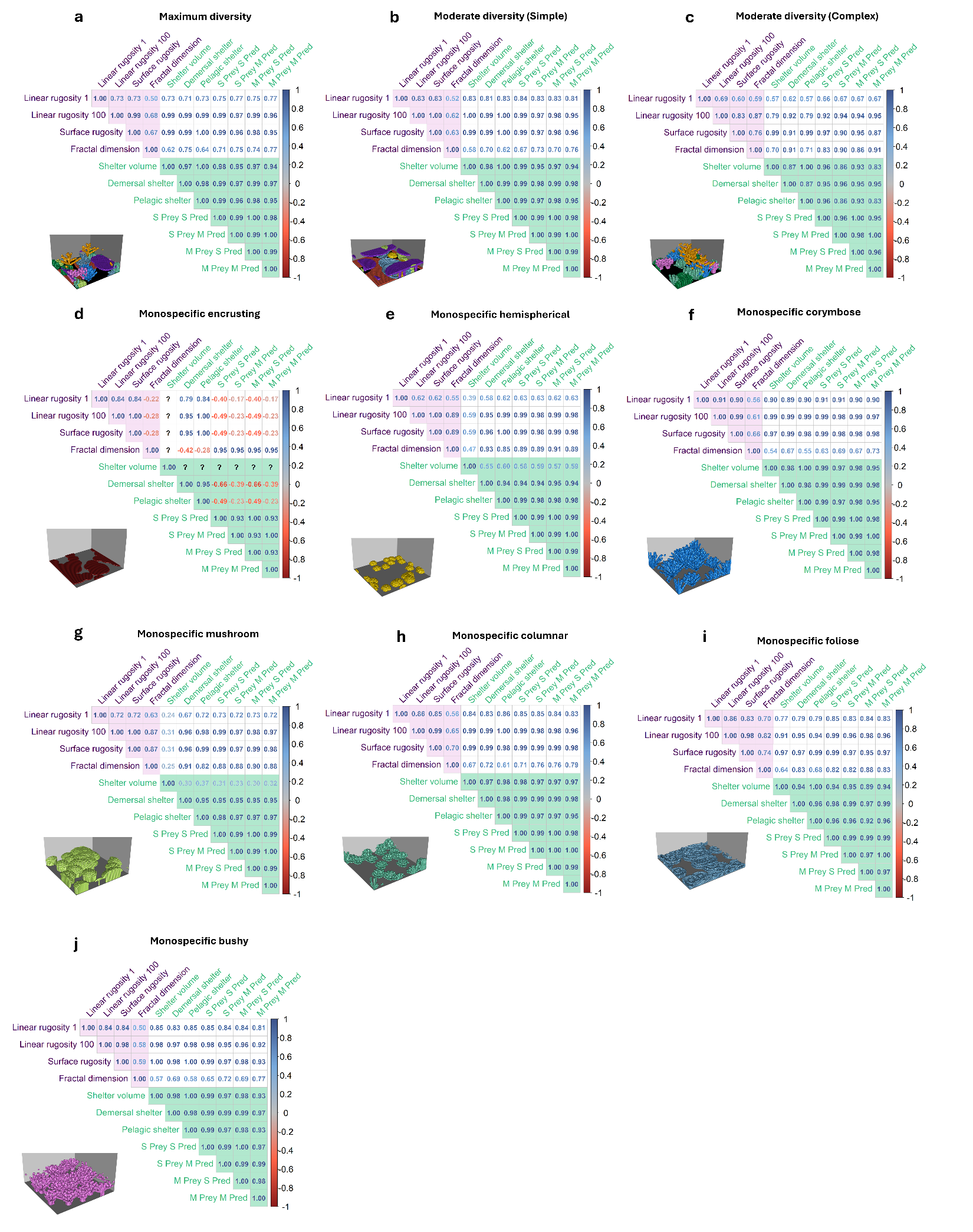


Fig. S1 Correlation matrices among structural (purple text) and shelter (green text) metrics calculated every 13 timesteps from 100 replicate simulations – correlations calculated for individual community types of a) maximum diversity, moderate diversity with b) simple and c) complex coral morphologies, monospecific d) encrusting, e) hemispherical, f) corymbose, g) mushroom, h) columnar, i) foliose and j) bushy community types. Numerical values represent the correlation value to two decimal places as calculated by Pearson’s correlation test. The colour of the numbers indicates the strength of correlation (red: negative correlation, blue: positive correlation, see colour gradient bar). Correlation value is only shown when significant (*p* < 0.05). Purple background shading indicates correlations among structural metrics, green background indicates correlations among shelter metrics, and correlations between structural and shelter metrics have unshaded background


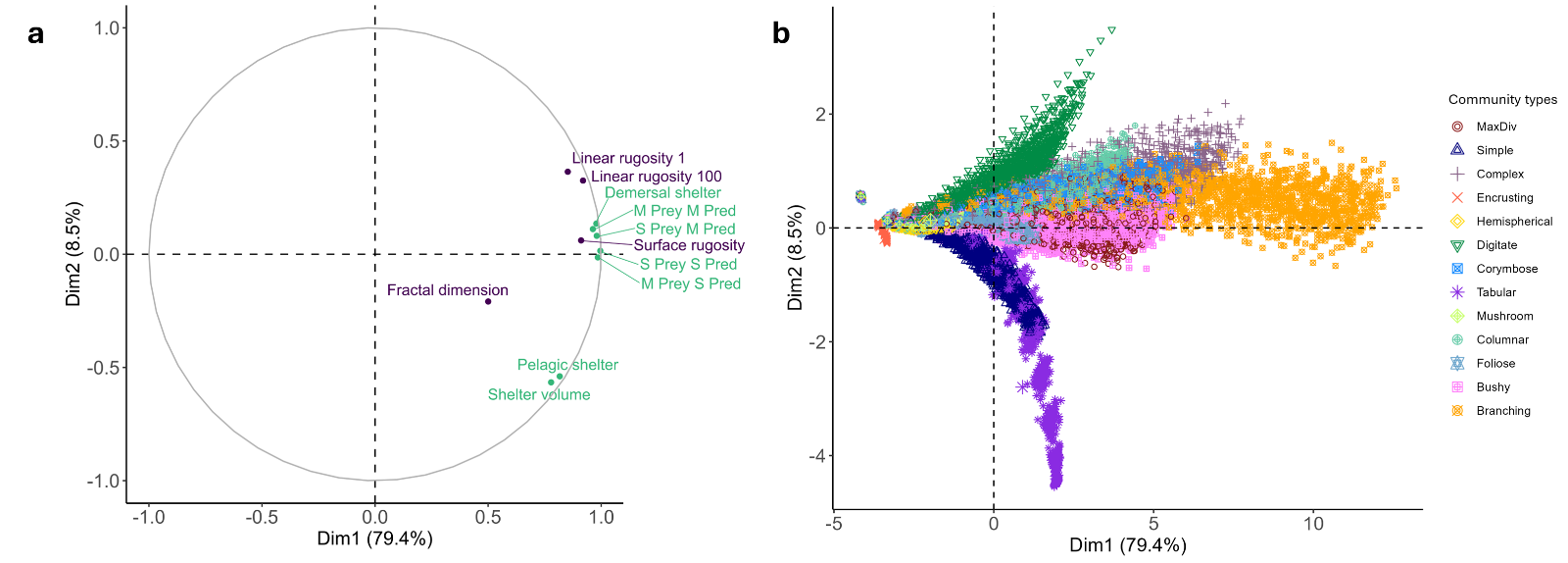


Fig S2 Principal component analysis (PCA) ordination of (a) 11 habitat complexity metrics and (b) individual observations calculated every 260 timesteps for all 100 simulations of data pooled together across 13 community types. Purple points and text indicate the four structural metrics, green points and text indicate the seven shelter metrics. See Oh et al., 2025a for detailed description of different coral community types.


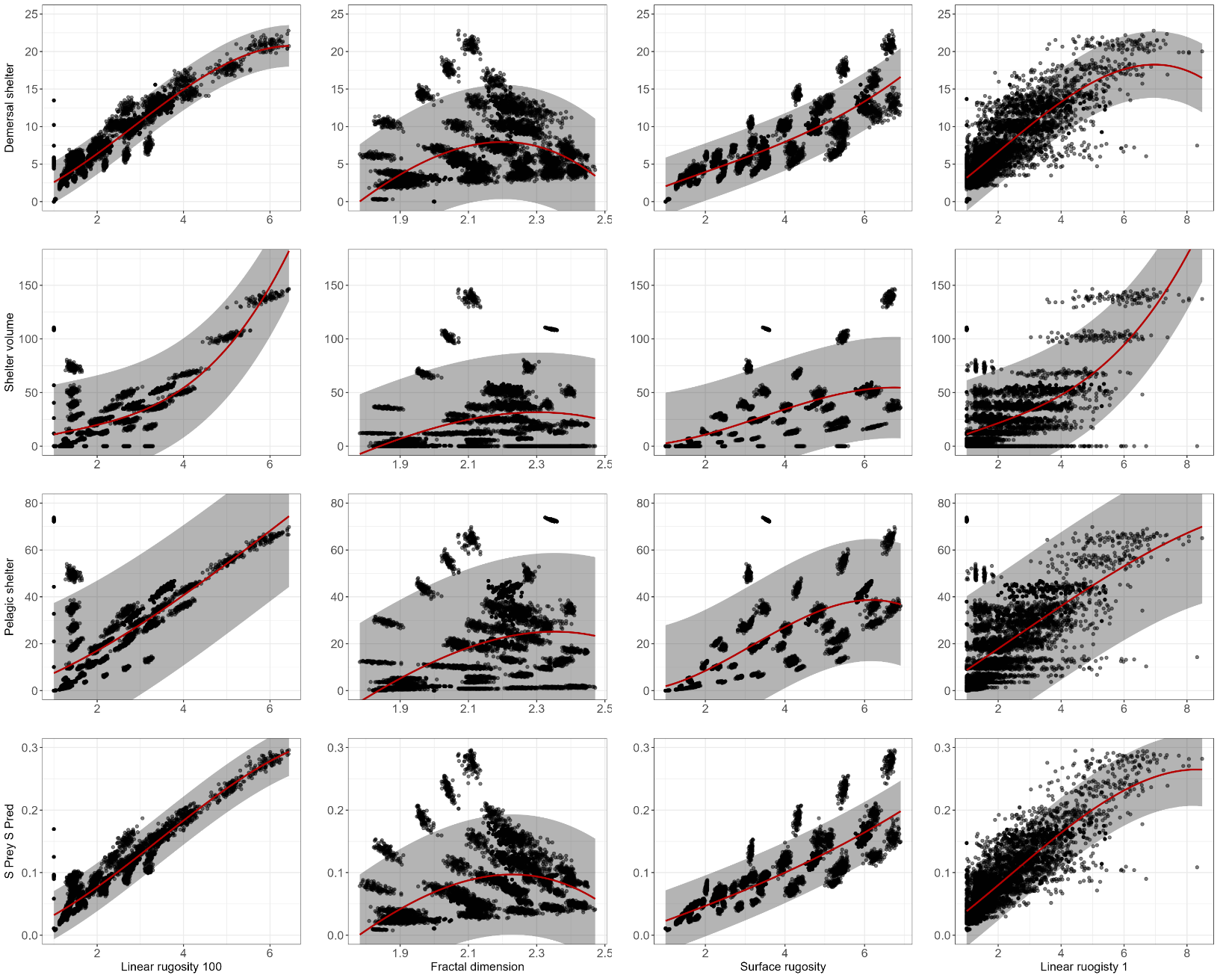


Fig. S3 Relationship between four structural (linear rugosity 100, fractal dimension, surface and linear rugosity 1, x-axis) and four shelter (shelter volume, demersal shelter, pelagic shelter and 1 combination of size-dependent shelter, y-axis) metrics calculated every 260 timesteps for all 100 simulations of data pooled together across 13 community types. Red line and grey ribbon show the mean and prediction interval from statistical cubic model added to aid pattern visualisation


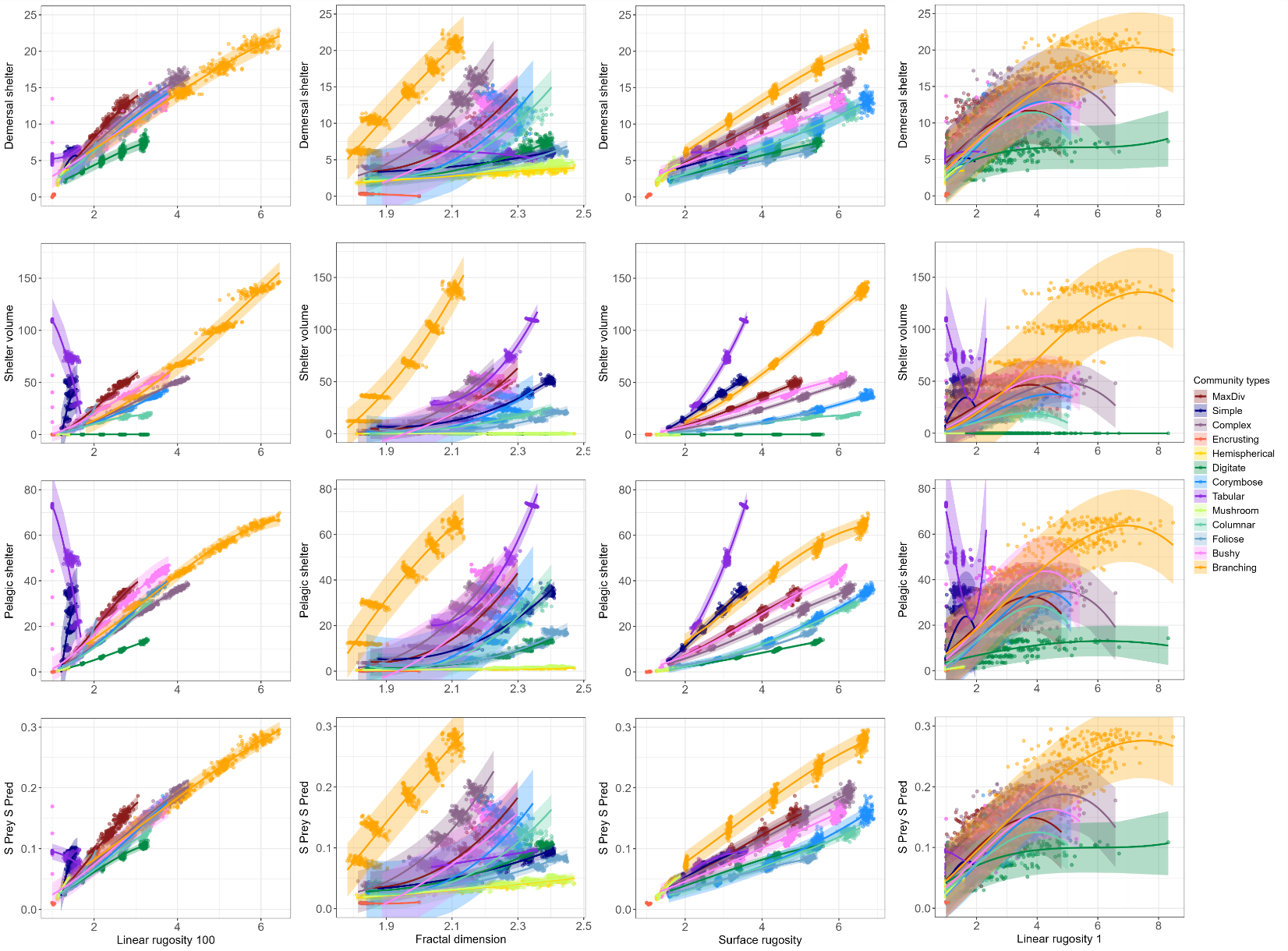


Fig. S4 Relationship between four structural (linear rugosity 100, fractal dimension, surface and linear rugosity 1, x-axis) and four shelter (shelter volume, demersal shelter, pelagic shelter and 1 combination of size-dependent shelter, y-axis) metrics calculated every 260 timesteps across all 100 replicates of each of the 13 community types. Coloured lines and ribbons show the mean and prediction interval from the statistical cubic models of the respective community type to aid pattern visualisation.


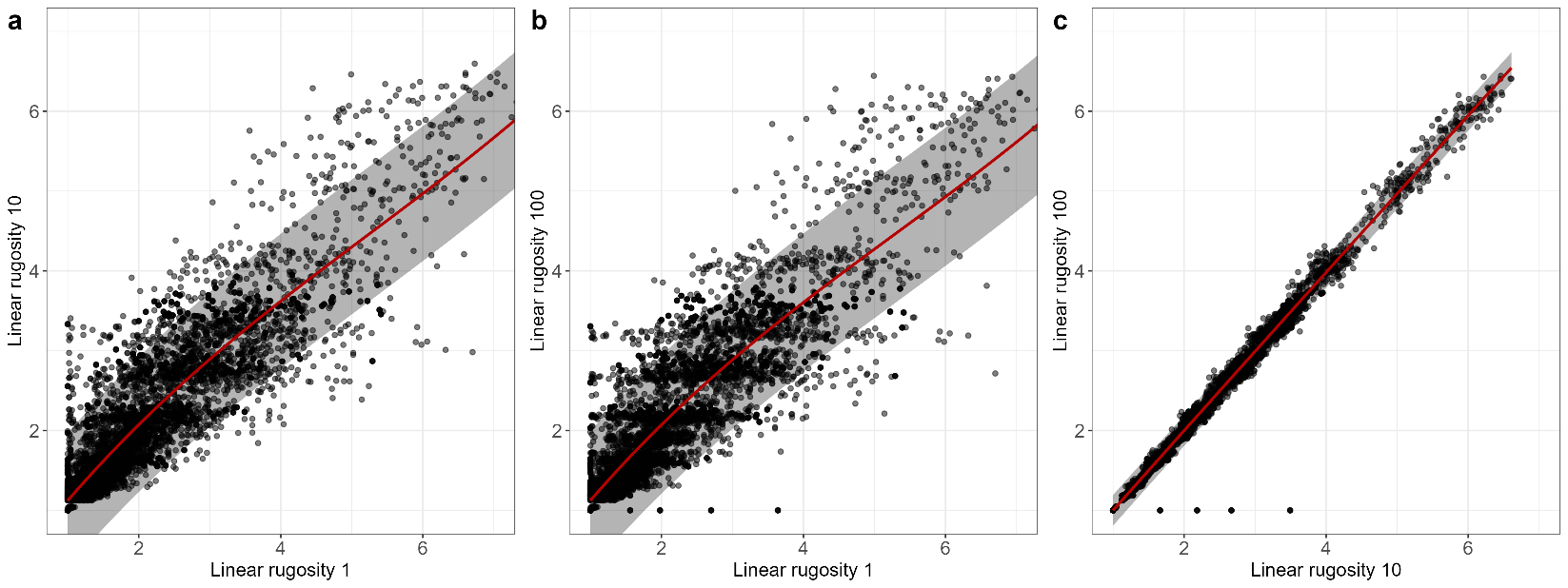


Fig. S5 Relationships between three linear rugosity metrics: (a) linear rugosity 1 and linear rugosity 10 (mean of ten linear rugosity 1), (b) linear rugosity 1 and linear rugosity 100, and (c) linear rugosity 10 and linear rugosity 100 for all 100 simulations of data pooled together across 13 community types. Red line and grey ribbon show the mean and 95% prediction interval from statistical cubic model added to aid pattern visualisation.
